# Characterization of placental endocrine function and fetal brain development in a mouse model of small for gestational age

**DOI:** 10.3389/fendo.2023.1116770

**Published:** 2023-02-10

**Authors:** Jorge Lopez-Tello, Amanda N. Sferruzzi-Perri

**Affiliations:** Centre for Trophoblast Research – Department of Physiology, Development and Neuroscience, University of Cambridge, Cambridge, United Kingdom

**Keywords:** placenta, mouse, pregnancy, prolactin, pregnancy specific glycoprotein, fetal growth, animal models, endocrinology

## Abstract

Conditions such as small for gestational age (SGA), which is defined as birthweight less than 10^th^ percentile for gestational age can predispose to neurodevelopmental abnormalities compared to babies with normal birthweight. Fetal growth and birthweight depend on placental function, as this organ transports substrates to the developing fetus and it acts as a source of endocrine factors, including steroids and prolactins that are required for fetal development and pregnancy maintenance. To advance our knowledge on the aetiology of fetal growth disorders, the vast majority of the research has been focused on studying the transport function of the placenta, leaving practically unexplored the contribution of placental hormones in the regulation of fetal growth. Here, using mice and natural variability in fetal growth within the litter, we compared fetuses that fell on or below the 10^th^ percentile (classified as SGA) with those that had adequate weight for their gestational age (AGA). In particular, we compared placental endocrine metabolism and hormone production, as well as fetal brain weight and expression of developmental, growth and metabolic genes between SGA and AGA fetuses. We found that compared to AGA fetuses, SGA fetuses had lower placental efficiency and reduced capacity for placental production of hormones (e.g. steroidogenic gene *Cyp17a1*, prolactin *Prl3a1*, and pregnancy-specific glycoproteins *Psg21*). Brain weight was reduced in SGA fetuses, although this was proportional to the reduction in overall fetal size. The expression of glucose transporter 3 (*Slc2a3*) was reduced despite the abundance of AKT, FOXO and ERK proteins were similar. Developmental (*Sv2b* and *Gabrg1*) and microglia genes (*Ier3*), as well as the pregnancy-specific glycoprotein receptor (*Cd9)* were lower in the brain of SGA versus AGA fetuses. In this mouse model of SGA, our results therefore demonstrate that placental endocrine dysfunction is associated with changes in fetal growth and fetal brain development.

## 1 Introduction

Abnormal birthweight is one of the most common complications of pregnancy that has immediate and long-term consequences for offspring wellbeing (1). Compared to babies that have an appropriate weight for their gestational age (AGA), babies born small (<10th percentile) or large for gestational age (>90th percentile) (SGA or LGA, respectively) are at higher risk of obstetric and neonatal complications (2, 3). Moreover, studies have shown that SGA or LGA increases the risk for metabolic diseases and cognitive and neurodevelopmental abnormalities compared to AGA (4–8). The identification and subsequent classification of babies with abnormal weight is challenging, as it requires segregation of babies that are constitutively small or large due to parental genetics versus babies that grew abnormally due to intrauterine problems (9–11). Therefore, work is required to understand the causes and mechanisms by which altered fetal growth may arise.

Fetal growth is regulated, in part, by the placenta which is a fetal organ responsible for the transport of gases and nutrients between the mother and the fetus. Aberrant placental function related to failure of substrate transport or deficits in vascular growth have been linked to fetal growth abnormalities, including fetal growth restriction, as well as stillbirth (12–14). However, the placenta is also a powerful endocrine organ that secretes an abundance of hormones and growth factors into the maternal and fetal circulation that allow for fetal development and pregnancy maintenance (15–20). Indeed, there is now growing evidence suggesting that placental metabolism and function impact on fetal brain development (21–27). For example, in mice, there is genomic linkage between placental and the fetal hypothalamic development (28). In mice, placental metabolism of serotonin is critical for fetal forebrain development. Others have found that placental allopregnanolone deficiency in mice alters cerebellar white matter development and programmes postnatal autistic-like behaviour in the offspring (24). Therefore, these studies suggest a potential link between placental endocrine function, fetal growth and fetal neurodevelopment, which could have long last effects on neurocognitive health outcomes.

In this scenario, animal models in which fetal and postnatal outcomes can be monitored are essential. Much of the investigations focused on fetoplacental development have been conducted in rodents, rabbits, sheep and pigs (29–36). However, the use of mice is still preferred over the aforementioned animal species due to multiple reasons. For example, the short gestational period and the relatively easy, lower cost maintenance of mice compared to large animal species is great for investigating fetal growth control. In mice, the sequence of events for brain maturation are largely similar to in humans (37). Moreover, a similar to in human, the mouse placenta is haemochorial in structure. An additional key advantage of the mouse is that its placenta is structurally divided in two functionally specialised regions; transport is carried out in the labyrinth zone, while hormone production is principally performed by the junctional zone (Jz). This characteristic is very helpful when studying placental function, as both layers can be easy separated (38). Nonetheless, the vast majority of published studies assessing the importance of the placenta for fetal growth have focused on placental transport function [e.g (31, 39–43).], leaving the significance of placental endocrine function practically unexplored.

Numerous animal models of pregnancy complications have been created, including those that mimic environmental and maternal conditions (11, 29, 36). However, models based on constructing fetal growth curves and percentile cut-offs, as applied in human obstetrics, are not commonplace for experimental research on placental function and fetal physiology (44). Here, using the mouse as an experimental animal model, we took advantage of the normal fetal weight variation observed within the litter to compare placental endocrine function and fetal brain development for fetuses that were classified as AGA or SGA using percentile cut-offs. We therefore hypothesize that abnormal placental endocrine output is an additional factor contributing to the development of SGA. Moreover, using fetal growth curves and percentile cut-offs in a model in which maternal health is preserved provides us with a valuable opportunity to study endocrine interaction between the placenta and fetus in a controlled intrauterine environment. Indeed, our work identifies that the production of prolactins and pregnancy specific glycoproteins (PSGs) by the placenta is compromised in conjunction with reduced fetal growth and brain development of SGA fetuses. Our work reinforces the idea of a placental-fetal brain axis that is controlled, in part, by placental hormones (25).

## 2 Materials and methods

### 2.1 Animal work

All animal work was performed under the UK Animals (Scientific Procedures) Act 1986. Experiments were conducted with a total of 11 C57BL6/J wild-type female mice (4 months old) housed at the University of Cambridge Animal Facility under a 12/12 dark-light system and fed *ad libitum* with a standard chow diet (RM3; Special Dietary Services). Females were time-mated with C57BL6/J males and the day a copulatory plug was detected was designated as gestational day (GD) 1 (term occurs ∼GD20). On GD16, pregnant females were killed by cervical dislocation, the uterus removed and fetuses cleaned from feto-placental membranes. Each fetus (and its corresponding placenta), was identified based on its position in the uterus, dried on tissue paper, weighed and immediately decapitated. Fetal brains were removed, weighed and immersed in liquid nitrogen for rapid freezing. The placental Jz was dissected from maternal decidua and placental labyrinth zone, and subsequently snap-frozen in liquid nitrogen. All frozen fetal and placental samples were stored at −80°C until tissues were powdered in individual pieces of foil using a hammer and dry ice for RNA/protein extraction (all samples were maintained in a frozen state, as far as possible). Only viable fetuses were used in the study and litter sizes ranged from 6 to 11 pups.

### 2.2 RNA extraction, cDNA reverse transcription and qPCR

Placental Jz and fetal brain RNA (8-9 samples per group) was extracted using the RNeasy Plus Mini Kit (Qiagen) and the quantity of RNA obtained was determined using a NanoDrop spectrophotometer (NanoDrop Technologies) as previously described (45). RNA was reverse transcribed using a high-capacity cDNA reverse transcription kit (Applied Biosystems) according to manufacturer’s instructions. Samples were analysed with a StepOne real-time PCR machine (Thermo Fisher Scientific) in duplicate using SYBR Green qPCR master mix (Applied Biosystems, Thermo Fisher Scientific) and primers described in **Supplementary Table 1**. Brain gene expression was normalized to the genomic mean of two housekeeping genes (*Actb* and *Gapdh*), while placental genes were normalized using *Actb* and *Ywhaz* as reference genes. These housekeeper genes remained stably expressed between the groups. Analysis was performed using the 2-ΔΔCt method (46).

### 2.3 Protein extraction and western blotting

Total protein was extracted from fetal brain and placental Jz (5 samples per group) using RIPA buffer (R0278-50M, Sigma Aldrich) supplemented with mini EDTA-free protease inhibitor cocktail mix (Roche), 1mM β-glycerophosphate (G-9891, Sigma Aldrich) and 1mM sodium orthovanadate (S65089891, Sigma Aldrich). Lysates were centrifuged at 2,500 rpm for 15 minutes at 4°C and protein concentration determined using the Pierce BCA protein assay kit (23225, Thermo Fisher Scientific). Samples were mixed with SDS gel loading buffer (L-4390, Sigma Aldrich) and protein denaturation was performed at 90°C for 5 minutes. After gel electrophoresis, proteins were transferred from the gel onto 0.45 µm nitrocellulose membranes (10600012, Amersham Protran). Membranes were then blocked either with 5% fetal bovine serum (A2153-100G, Sigma Aldrich) or semi-skimmed milk (Marvel) for 1 hour at room temperature and incubated overnight at 4°C with primary antibodies described in **Table 1**. The day after, membranes were washed with TBS-T and incubated for 1 hour at room temperature with secondary antibodies (1:10,000 NA934 or NA931, Amersham). Membranes were exposed to ECL substrate (SuperSignal West Femto, Thermo Fisher Scientific) and images were taken with an Invitrogen iBright imaging system (Thermo Fisher Scientific). Pixel intensity of protein bands was analysed with ImageJ software and normalization performed against beta-actin levels. Phosphorylated proteins were normalized to its corresponding total protein abundance.

**Table 1:** List of antibodies used for western blotting.

Protein	Catalogue number	Dilution
Insulin receptor	Santa Cruz, SC-711	1/200
AKT	Cell Signalling, 9272	1/1,000
Phospho-AKT (Ser473)	Cell Signalling, 9271	1/1,000
Phospho-AKT (Thr308)	Cell Signalling, 9275	1/1,000
mTOR GβL	Cell Signalling, 3274	1/1,000
FOXO1 (C29H4)	Cell Signalling, 2880	1/1,000
Phospho-FOXO1 (Ser256)	Cell Signalling, 9461	1/1,000
FOXO3a (D19A7)	Cell Signalling,12829	1/1,000
Phospho-FOXO3a (Ser318/321)	Cell Signalling, 9465	1/1,000
P44/42 MAPK (Erk1/2)	Cell Signalling, 4695	1/1,000
Phospo-MAPK-p44/42 (Erk1/2) (Thr202/Tyr204)	Cell Signalling, 4370	1/1,000
Beta-Actin	Cell Signalling, 58169	1/5,000

### 2.4 Sample size and statistical analysis

No sample size calculation was conducted prior to undertaking this study. For the purpose of this paper, the fetus, and not the mother, was designated as the experimental unit. GraphPad Prism software (version 9) was used to determine statistical differences between groups. The distribution of fetal weights (graphed as a histogram) was constructed with a non-linear regression (Gaussian distribution) and percentiles calculated with the descriptive statistical analysis tool of GraphPad Prism software. Then, data in Excel were organized according to fetal weight and separated in three experimental groups based on the fetal weight threshold (SGA fetuses: 10^th^ percentile ≤321 mg; AGA fetuses: 321-409 mg and LGA fetuses: 90^th^ percentile≥410 mg). ROUT test was used to identify outlier values, which were then excluded for statistical purposes. Normality and homogeneity of variance of variables for western blotting and qPCR data were performed using the Shapiro-Wilks test in GraphPad Prism. For comparisons between two groups, the Student’s t-test or Mann-Whitney test were applied according to the normality of the variable. For data with more than two groups, one-way ANOVA coupled with Bonferroni *post hoc* test was employed. The relation between fetal intrauterine position as well as litter size with the presence of SGA, AGA and LGA was analysed by Chi square test using a contingency table in GraphPad Prism software. Pearson r correlations were performed with GraphPad Prism software and presented in a double gradient colour mapping graph that was subsequently modified with Adobe Illustrator to facilitate visualization. qPCR and western blotting data are expressed as individual data points and reported as mean ± SEM. P*-*values <0.05 were considered statistically significant.

## 3 Results

### 3.1 Validation of the SGA model

**Figure 1:**
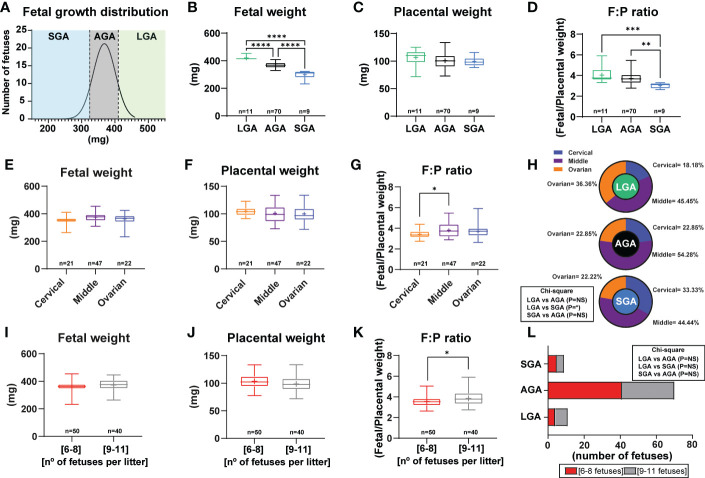
Fetal and placental weight in mice and establishment of a model to study small for gestational age. **(A)** Frequency distribution curve for fetal weights on GD16. Vertical line denotes the 10^th^ (321mg) and 90^th^ percentiles (410 mg). **(B)** Fetal weight for fetuses classified as SGA, AGA and LGA. **(C)** Placental weight classified as SGA, AGA and LGA. **(D)** Placental efficiency (defined as grams of fetus produced by grams of placenta) classified as SGA, AGA and LGA. **(E–G)** Feto-placental weights and placental efficiency based on their position in the uterus. **(H)** Distribution of LGA, AGA and SGA fetuses according to their position in the uterus. **(I–K)** Feto-placental weights and placental efficiency based on litter size. **(L)** Distribution of LGA, AGA and SGA fetuses according to litter size. Data are from 90 fetuses in total from 11 litters. Feto-placental weights are shown as box plots and whiskers. The rectangle shows the distribution, the line the median, the whiskers the maximum and minimum and the “+” is the mean of the group. Statistical analysis performed by one-way ANOVA, Student t-test, Mann-Whitney test and Chi-square test. *P<0.05, **P<0.01, ***P<0.001, ****P<0.0001. AGA (appropriate for gestational age), LGA (large for gestational age), SGA (small for gestational age), n° (number).

The distribution of fetal weights across the 11 litters studied is shown in **Figure 1A** and litter sizes across the different litters used in this study can be found in **Supplementary Table 2**. A total of 9 fetuses were considered SGA (below the 10^th^ percentile) and they were obtained from 5 different litters. On average, fetal weight was significantly lower by 19% in SGA fetuses when compared to AGA fetuses (**Figures 1A, B**). A total of 11 LGA fetuses (above the 90^th^ percentile) were obtained from 3 different litters and these were on average, 12% heavier than the AGA group. All litters had at least 3 AGA fetuses. Analysis of placental weight did not show significant differences between the SGA, AGA and LGA fetuses (**Figure 1C**). However, the fetal weight to placental weight ratio, defined as grams of fetus produced by grams of placenta and also known as placental efficiency, was significantly reduced in the SGA fetuses compared to AGA and LGA fetuses. No differences in placental efficiency were observed between the AGA and LGA fetuses (**Figure 1D**).

Fetal and placental weights were not affected by the uterine position, as similar weights were found for fetuses positioned close to the ovary, cervix, and in between these two positions (**Figures 1E, F**). Nonetheless, placental efficiency was significantly lower by 10% for fetuses positioned close to the ovary compared to those in the middle of the uterus (**Figure 1G**). Individual group analysis demonstrated that there was not a clear pattern in the incidence of being SGA, AGA or LGA based on uterine position. Although a significant effect was observed between fetal position and being LGA or SGA (**Figure 1H**). Indeed, the number of SGA fetuses positioned close to the cervix was higher than for those proximal to the ovary (33.33% versus 22.22%; **Figure 1H**). This pattern was the opposite for LGA fetuses, with 22.22% located close to the cervix and 44.44% close to the ovary. AGA fetuses were primarily found in the middle part of the uterus (54.28%).

Finally, we divided our data according to the number of fetuses per litter to assess if litter size impacted the percentage of SGA, AGA and LGA (litters of 6 to 8 fetuses versus 9 to 11 fetuses; **Figures 1I–L**). This analysis revealed that there was no difference between fetal or placental weight, nor the proportion of fetuses classified as SGA, AGA or LGA between litters of 6 to 8 fetuses compared to those of 9 to 11 fetuses (**Figure 1L**). However, placental efficiency was significantly greater by 7.80% in litters with 9 to 11 fetuses compared to those with 6 to 8 (**Figure 1K**). Moreover, we then performed analysis of LGA and SGA fetuses by considering the litter size as a potential effect (comparison of LGA-SGA fetuses in litters of 6-8 fetuses versus LGA-SGA fetuses in litters of 9-11 fetuses). As displayed in **Supplementary Table 3**, we did not see differences in LGA or SGA fetuses in any of the aforementioned parameters.

### 3.2 Placental endocrine function in SGA fetuses

**Figure 2:**
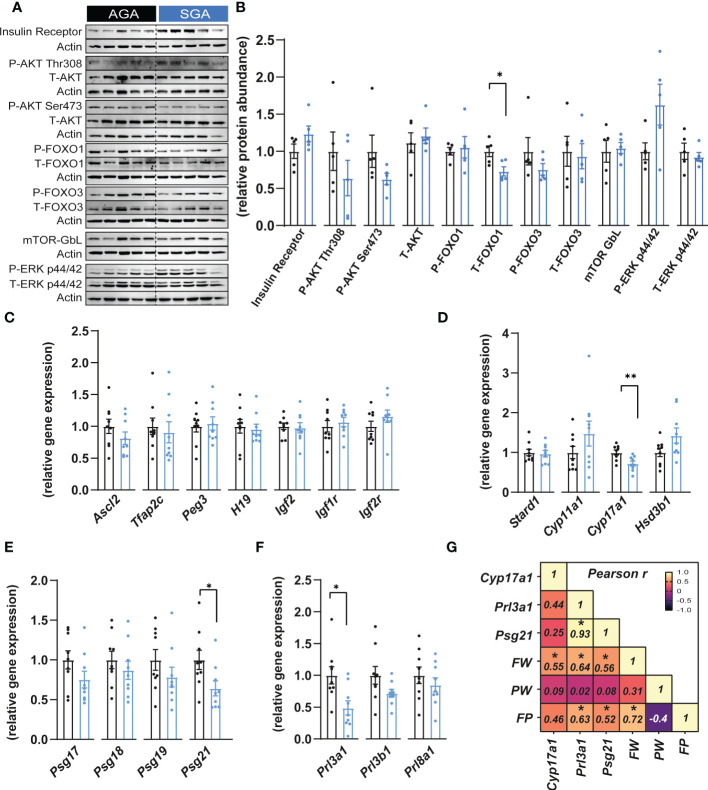
Small for gestational age is associated with altered expression of placental hormones. **(A, B)** Immunoblots and protein abundance of key growth and metabolic signalling proteins in dissected placental junctional zones (n=5 samples per group). **(C)** Expression of key genes involved in placental function and growth (n=9 samples per group). **(D–F)** Expression of steroidogenic pathway regulatory **(D)**, pregnancy specific glycoprotein **(E)** and prolactin **(F)** genes in dissected placental junctional zones (n=9 samples per group). **(G)** Heatmap of Pearson’s correlations (number inside the box corresponds to the r value for the correlations that were significant) (n=18 samples). Data are shown as individual values and bars represent mean ± SEM. Statistical analysis performed by paired or unpaired t-test (Student t-test and Mann-Whitney test, respectively). *P<0.05, **P<0.01. AGA (appropriate for gestational age) and SGA (small for gestational age), FW (fetal weight), FP (feto-placental ratio, also known as placental efficiency), PW (placental weight), P (phosphorylated protein levels), T (total protein levels).

To understand if the reduced placental efficiency for SGA fetuses was related to alterations in the endocrine function, key growth and metabolism proteins were quantified by western blotting in the placental Jz of SGA compared to AGA fetuses (**Figures 2A, B**). This revealed that the abundance of FOXO1, which functions in cell cycle and differentiation (47), was significantly reduced by 27% in the SGA compared to AGA group without affecting phosphorylated site at serine 256 (ratio of phosphorylated to total FOXO1; **Figure 2B**). However, the abundance the insulin receptor β, AKT (total and phosphorylated at threonine 308 and serine 473 sites), FOXO3 (total and phosphorylated at serine 318/321 sites), ERK (total and phosphorylated at threonine 202 and tyrosine 204 site) and mTOR- GβL were unaltered between SGA and AGA fetuses (**Figures 2A, B**).

We next evaluated whether the expression of imprinted genes could be altered in placental Jz samples from SGA fetuses as previous studies have shown that, in mice, the imprinted loci *Igf2-H19, Ascl2* and *Peg3* are implicated in the control placental Jz formation and/or function (48–51). No changes were observed in any of the imprinted genes analysed in the Jz between SGA and AGA (**Figure 2C**). The Jz expression of *Tfap2c*, which has been shown to control the expression of certain imprinted genes such as *H19* and *Ascl2* (52), was also unaltered between the two experimental groups (**Figure 2C**). Moreover, mRNA levels of the IGF2 receptors, *Igf1r* and *Igf2r* remained similarly expressed in the Jz between SGA and AGA (**Figure 2C**).

We then assessed the placental capacity to produce hormones by measuring mRNA levels of genes involved in steroidogenesis (*Stard1*, *Cyp11a1*, *Cyp17a1* and *Hsd3b1*), pregnancy specific glycoprotein genes (PSGs; *Psg17*, *Psg18*, *Psg19* and *Psg21*) and prolactins (*Prl3a1*, *Prl3b1* and *Prl8a1*) in the Jz of SGA and AGA fetuses (**Figures 2D–F**). We observed that *Cyp17a1* expression was 28% lower in the placental Jz of the SGA group compared to AGA (**Figure 2D**). Moreover, *Psg21* mRNA levels were reduced by 36% in the SGA versus AGA fetuses (**Figure 2E**). The expression of prolactin genes, *Prl3a1* was also downregulated by 51% in the SGA group versus the AGA (**Figure 2F**). However, the Jz expression of other hormone-related genes quantified, namely *Stard1*, *Cyp11a1*, *Hsd3b1, Psg17, Psg18, Psg19, Prl3a1, Prl3b1* and *Prl8a1* were not different between the two experimental groups. To understand the contribution of changes in placental Jz *Cyp17a1, Psg21* and *Prl3a1* expression for feto-placental size and placental efficiency, we performed Pearson r correlations (**Figure 2G**). This revealed that Jz expression of all three genes correlated positively with fetal weight. *Prl3a1* and *Psg21* correlated positively with placental efficiency. Both, *Prl3a1* and *Psg21* correlated positively with each other. Lastly, placental efficiency, but not placental weight correlated positively with fetal weight (**Figure 2G**).

### 3.3 Brain development in SGA fetuses

**Figure 3:**
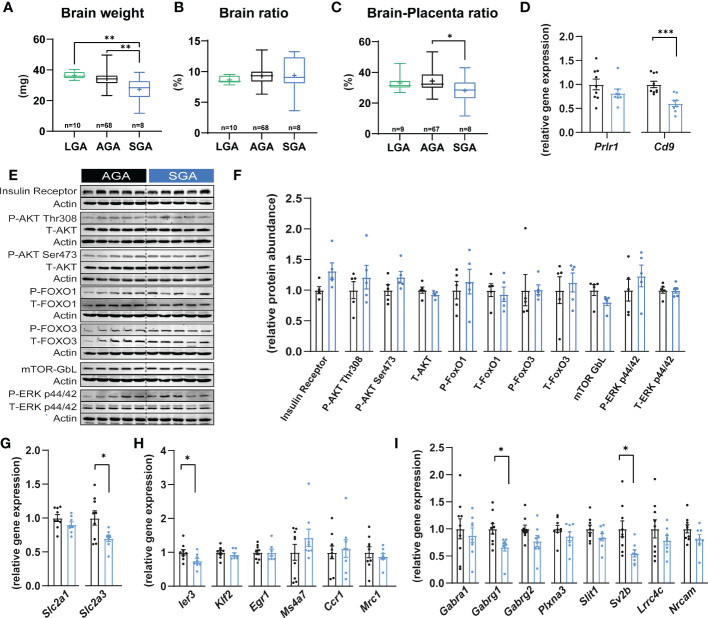
Small for gestational age and its impact on fetal brain. **(A)** Brain weight. **(B)** Brain weight expressed as a ratio of the brain weight divided by fetal weight. **(C)** Brain weight expressed as a ratio of brain weight divided by placental weight. **(D)** The expression of key prolactin and pregnancy-specific glycoprotein receptors in fetal brain (n=8-9 samples per group). **(E, F)** Immunoblots and protein abundance of key growth and metabolic signalling proteins in fetal brain (n=5 samples per group). **(G–I)** Expression levels of glucose transporters, microglia and axon developmental genes (n=8-9 samples per group). Data are from 90 fetuses in total from 11 litters (excluding outliers). Data are shown as individual values and bars represent mean ± SEM. Statistical analysis performed by one-way ANOVA followed by Bonferroni *post hoc* test (variables of three groups), paired or unpaired t-test (Student t-test and Mann-Whitney test, respectively). ROUT test was conducted to detect and eliminate outliers. *P<0.05, **P<0.01, ***P<0.001. AGA, (appropriate for gestational age); SGA, (small for gestational age); P, (phosphorylated protein levels); T, (total protein levels).

Short and long-term neurological impairments have been observed in offspring who were SGA (53). Therefore, we investigated if there could be any changes in fetal brain development in our mouse model of SGA. We found that SGA fetuses exhibited a 20% and 25% reduction in brain weight compared to the AGA and LGA groups, respectively (**Figure 3A**). In contrast, brain weight was similar between AGA and LGA fetuses (**Figure 3A**). Analysis of fetal brain weight relative to fetal body size revealed that the reduction in brain size was proportional to the reduced size of the SGA fetuses (brain to body weight ratio; not different between SGA, AGA and LGA fetuses; **Figure 3B**), which suggested that SGA fetuses were symmetrically smaller. We then determined the relationship between placental and fetal brain size by assessing the ratio of fetal brain weight to placental weight. This showed that fetal brain weight to placental weight ratio was significantly reduced by 18% in SGA compared to AGA fetuses (**Figure 3C**). However, this ratio was not different between LGA and AGA fetuses.

We also analysed the effect of uterine position and litter size on fetal brain development (**Supplementary Figure 1**). We did not see differences in the weight of the brain, although brain ratio was significantly increased in fetuses positioned closer to the ovaries compared to those in the middle position (**Supplementary Figures 1A, B**). No differences were found in the brain-placental ratio and litter size did not affect the weight of the brain, although brain ratio was significantly increased in litters of 6-8 fetuses compared to those of 9 to 11 fetuses (**Supplementary Figure 1C–F**). Analysis of LGA and SGA fetuses by considering the litter size as a potential effect (comparison of LGA-SGA fetuses in litters of 6-8 fetuses versus LGA-SGA fetuses in litters of 9-11 fetuses) showed that LGA fetuses in litters of 6-8 fetuses had reduced brain ratio and brain-placental ratio compared to those LGA in larger litters (**Supplementary Table 3**). These effects were not found in the SGA fetuses (**Supplementary Table 3**).

To further investigate the relationship between placental function and fetal brain we quantified the mRNA levels of receptors that mediate the action of prolactins and PSGs, *Prlr1* and *Cd9*, respectively in SGA compared to AGA fetuses (54, 55). We found that the expression of *Cd9* was 39.5% lower in the brain of SGA versus AGA fetuses (**Figure 3D**). However, the mRNA expression levels of *Prlr1* in the brain were similar between SGA and AGA fetuses. To understand the potential molecular changes underlying brain weight differences in SGA versus AGA fetuses, we quantified the levels of key cellular signalling pathways involved in growth and metabolism and found that these remained similar in the brain of SGA and AGA fetuses (e.g. insulin receptor β, AKT, FOXO1-3, mTOR- GβL and ERK; **Figures 3E, F**). As the fetal brain is highly dependent on glucose metabolism for growth, we quantified the expression of genes encoding the main glucose transporters in the brain of SGA and AGA fetuses (56). Glucose transporter number 1 (*Slc2a1*) did not change between SGA and AGA fetuses; however, the mRNA levels *Slc2a3* were significantly reduced by 30% in SGA compared to AGA fetuses (**Figure 3G**).

We then explored whether our model of SGA and placental malfunction may be linked to changes in the differentiation and maturation of microglia. We analysed mRNA levels of 6 microglia markers (*Ier3*, *Klf2*, *Egr1*, *Ms4a7*, *Ccr1* and *Mrc1*) (57) and found that 5 out of 6 were stably expressed in the brain of SGA compared to AGA fetuses (**Figure 3H**). Interestingly, the expression of *Ier3* was significantly reduced by 25% in SGA compared to AGA fetuses. We then quantified the expression of 8 genes involved in axonogenesis (58) (*Plxna3, Slit1, Gabra1, Sv2b, Gabrg1, Gabrg2*, *Lrrc4c* and *Nrcam*). We found that *Sv2b* and *Gabrg1* were reduced by 45% and 12% in SGA compared to the AGA group, respectively (**Figure 3I**). The other axonogenesis genes analysed did not differ between SGA and AGA.

## 4 Discussion

In this study we have shown that the defects in placental endocrine function are associated with reduced fetal growth and brain development in a mouse model of SGA. In particular, we found that the placental capacity to respond metabolically (FOXO1) and produce sex steroids (*Cyp17a1)*, prolactins (*Prl3a1*) and PSGs (*Psg21*) were compromised in fetuses classified as SGA. Moreover, SGA fetuses have a proportional reduction in brain weight, low expression of glucose transporter 3 (*Slc2a3*), axogenesis (*Sv2b* and *Gabrg1*) and microglia (*Ier3*) genes along with reduced expression of the PSG receptor (*Cd9*) in the fetal brain of SGA fetuses compared to AGA. Finally, we found a significant link between the placenta and fetal brain development, as shown by the reduced brain-placental weight ratio in SGA fetuses. Together, our data reveal that placental hormones could be additional regulators of fetal growth with implications for fetal brain development. Furthermore, mis-communication of placental hormones with the developing fetal brain may have immediate and long-lasting implications for neurocognitive outcomes.

In the mouse, we have recently shown that the placental secretome comprises more than 1,000 proteins, including prolactins (17). In rodents, the prolactin family consists of 23 closely related genes and their expression varies spatially and temporally in the placenta (59). Although the role of prolactins in modulating maternal physiology is well-defined, for instance in metabolic adaptations during pregnancy (15, 60, 61) and in promoting maternal nurturing behaviour and lactation (62), their role in embryo and fetal development is not fully understood. In humans, prolactin can be found in the fetal circulation from mid-gestation and its receptors are expressed in fetal tissues by 7.5 weeks of gestation (63). In mice, prolactin receptors are widely expressed by the fetus, including its brain, with *Prlr* mRNA levels detectable as early as on GD8 (64). Prior work has speculated that prolactin in the fetus may be important for the growth of the adrenal cortex, production of pulmonary surfactant and in the control of the immune system (65, 66). However, further research is required to understand how the prolactin hormone family drives fetal growth, especially the growth and development of the fetal brain, given that placental production of PRL was decreased in our SGA mouse fetuses. Consistent with our findings, in humans, there is a down-regulation of the growth hormone/chorionic somatomammotropin (*hGH/CSH*) cluster in the SGA placenta (67). Moreover, other work has shown that mothers carrying SGA male fetuses display lower concentrations of prolactin in their circulation when compared to women carrying AGA male fetuses (68).

In our mouse model, the expression of *Cyp17a1* was lower in the SGA *versus* AGA fetuses. Our findings are consistent with studies in humans that show polymorphisms in *Cyp17a1* can influence the risk of SGA (69). In humans, the expression of *Cyp17a1* is mainly localized in the syncytiotrophoblast, acting as a source of estrogen during pregnancy (70). In mice, placental *Cyp17a1* expression in the placenta is modulated by *Igf2* (48); however in the present study, *Igf2* and other imprinted genes that regulate placental endocrine capacity were not differentially expressed between SGA and AGA fetuses. Other work has indicated that sex steroids interact with FOXO1 signalling (71). In addition, FOXO1 is important for embryonic development and placental differentiation (72). Thus, understanding the link between reduced FOXO1 abundance and decreased placental endocrine capacity in our SGA mouse model would be valuable.

The expression of PSGs was also altered in our mouse model of SGA, with lower *Psg21* mRNA levels in the placental Jz and this was couple to decreased fetal brain expression of the PSG receptor, *Cd9*. In rodents, PSGs are synthesised by spongiotrophoblasts and giant cells, while in humans, PSGs are produced by the syncytiotrophoblast (73–75). PSGs belong to the carcinoembryonic antigen family (76) and although the functions of PSGs are not fully elucidated, they are proposed to modulate immune cells (77, 78) and angiogenesis (79). Moreover, low levels of PSGs are associated with the development of pregnancy complications, including early pregnancy loss, placental insufficiency, fetal growth restriction and fetal hypoxia (80–84). Reinforced by our findings that placental *Prl3a1* and *Psg21* expression are positively correlated with fetal weight, future work should evaluate circulating levels of these hormones in fetal plasma. The placental transport labyrinth region in the mouse expresses receptors for such hormones (49, 85). Thus, future studies should evaluate whether the relationship between placental hormone production and fetal growth may also be mediated by potential local changes in the formation and function of the placental labyrinth as a result of altered paracrine signalling.

An additional objective of our work was to characterize the potential changes occurring in the fetal brain in our mouse SGA model. We did see a symmetrical reduction in brain weight and decreased mRNA levels of the glucose transporter 3 (*Slc2a3*) and impaired axogenesis markers (as inferred by the low expression levels of *Sv2b* and *Gabrg1*) in SGA fetuses. Of note, we did not find any changes in PI3K-AKT-mTOR signalling, which is critical for biological processes like nutrient uptake, cell growth and migration (86), in the brain of SGA compared to AGA fetuses. Changes in the PI3K-AKT-mTOR signalling cascade have been linked to microcephaly (87), due to reductions in neuronal cell division (88). We also did not identify any changes in FOXO1-3 and ERK signalling, and microglia markers (57) were similarly expressed between SGA and AGA fetuses, aside from the reduced mRNA levels of *Ier3*. Microglia are important specialised cell types in the nervous system that influence brain development, including neuronal proliferation or synaptic remodelling (89). SGA is a condition that can be linked to inflammation, as SGA newborns can exhibit elevated interleukin (IL) IL-1β, IL-8 and tumor necrosis factor in their circulation during the first postnatal weeks (90). The gene *Ier3* responds to changes in toll-like receptors (TLR3) (91) and its expression can be induced by different stimuli, including cytokines, infections and growth factors (92). In mice, low *Ier3* levels results in abnormal immune regulation and inflammation (92). Therefore, future work should explore the contribution of differences in *Ier3* in SGA fetus to the immune status, function and structural development of the developing brain. Regarding the changes in axogenesis markers, in the current study we employed whole brain lysates for our analyses and therefore cannot exclude the possibility of molecular changes in specific regions of the brain in the context of SGA. Nevertheless, in our SGA model, the expression of *Sv2b*, which encodes a synaptic vesicle protein and *Gabrg1*, which functions as a γ-Amino-butyric acid (GABA) receptor was reduced. Prior work has found that *Sv2b* is regulated by the maternal gut microbiota during mouse fetal brain development (58). This gene is broadly expressed in the central nervous system, with especially high expression in glutamatergic neurons (93, 94). Other investigations in mice, have also shown that intrauterine growth restriction is related to postnatal memory deficits that may be linked to abnormal proportions of maturing glutamatergic neurons at birth (95). Hence, study of the postnatal neurocognitive outcomes in our mouse model of SGA is warranted. In our study, the reduction in *Sv2b* and *Gabrg1* was in line with decreased expression of *Cd9* by the fetal brain in SGA fetuses, which may suggest a role for placental PSGs in shaping fetal axogenesis. Indeed, previous work in mice and humans has found CD9 is present in different cell types including astrocytes, sympathetic neurons and Schwann cells (96). Given that placental PSG production was also decreased in our SGA model, a greater understanding of how the placenta may influence fetal brain development through PSG signalling should be explored in future work.

A major limitation of the current study was that our study was not sufficiently powered to evaluate the influence of fetal sex on placental endocrine capacity and fetal brain development. This is important as previous work has demonstrated that fetal sex is an important determinant of placental endocrine capacity (48, 49). Furthermore, recent work in mice has revealed that placental steroid metabolism and metabolic signalling capacity differ depending on the size and the sex of the fetus (40). Moreover, the potential contribution of the labyrinth zone in the placental endocrine support of fetal development was not explored. This would be important in future work, as previous work has shown that the rodent labyrinth zone can assist in the production of steroids by the placenta (97). Moreover, steroids regulate glucose uptake through changes in *Slc2a1* and *Slc2a3* (98) and in our model we found reduced levels of *Slc2a3* in the SGA fetal brain. Moreover, the mouse is a litter-bearing specie and despite that our analysis of fetal intrauterine position and litter size composition show that there are no significant differences in fetal-placental weights (including fetal brain weight), we observed that placental efficiency is affected by both, the intrauterine fetal position and the number of fetuses per litter. Previous work performed in rats have identified greater blood flow at the cervical and ovarian ends compared to middle of the uterus, which may partly explain our findings (99). Work in multiple species has also found that placental efficiency is greater in monotocous species with multiple gestations and in polytocous species with larger litters, which is not surprising given the higher fetal demand on maternal resources (100, 101). Therefore, the analysis in combination of both, endocrine and vascular/transport function of the placenta, will provide a more accurate and detailed angle to understand the causes and short/long-term consequences of SGA.

In the current study, we intentionally studied unmanipulated, wildtype litters using fetal growth curves and percentile cut-offs to preserve maternal health and precisely study the role of placental endocrine interactions with the fetus in a controlled intrauterine environment. However, in doing so, we likely limited our ability to detect important pathways and proteins involved in placental endocrine function. Nonetheless, our work provides new evidence about the importance of placental hormones in the regulation of fetal growth and development. Moreover, the low levels of *Cd9* in the fetal brain suggest a direct link to changes in placental PSG production and sensitivity in the context of SGA. Further work is required to ascertain the specific contribution of the dysregulated placental hormones found in our SGA model (*Cyp17a1*, *Prl3a1*, *Prl3b1* and *Psg21*) and brain development. Moreover, animal studies deploying *in vivo* approaches to specifically delete genes in mouse placenta endocrine cells would be highly useful for decoding the communication between placental hormones and fetal organogenesis (48, 49, 102). This work could be complemented by experiments using fetal brain explants or cerebral organoids that are cultured with different combinations of placental hormones. To sum up, our results identify placental hormones as key regulators of fetal growth and may have relevance for understanding the aetiology of fetal growth disorders, and the mechanistic basis of associations between poor fetal growth and the subsequent increased risk of poor neurocognitive outcomes.

## Data availability statement

The data presented in the study are deposited in the Gene Expression Omnibus repository, accession number GSE224682.

## Ethics statement

The animal study was reviewed and approved by the United Kingdom Home Office under the Animals (Scientific Procedures) Act 1986 and underwent review by the University of Cambridge AnimalWelfare and Ethical Review Body.

## Author contributions

JL-T performed research. JL-T and AS-P wrote the paper and designed the study. All authors contributed to the article and approved the submitted version.

## Supplementary material

Supplementary Figure 1 Brain phenotype based on the intrauterine position and litter size. Data are from 90 fetuses in total from 11 litters (excluding outliers). Data are shown as box plots and whiskers. The rectangle shows the distribution, the line the median, the whiskers the maximum and minimum and the “+” is the mean of the group. Statistical analysis performed by one-way ANOVA followed by Bonferroni *post hoc* test (variables of three groups), paired or unpaired t-test (Student t-test and Mann-Whitney test, respectively). *P<0.05.
