# Supplementary figures and images for: Characterization of placental endocrine function and fetal brain development in a mouse model of small for gestational age
Source: Front Endocrinol (Lausanne). 2023 Feb 10;14:1116770. doi: 10.3389/fendo.2023.1116770 (PMC9950515; doi:10.3389/fendo.2023.1116770)

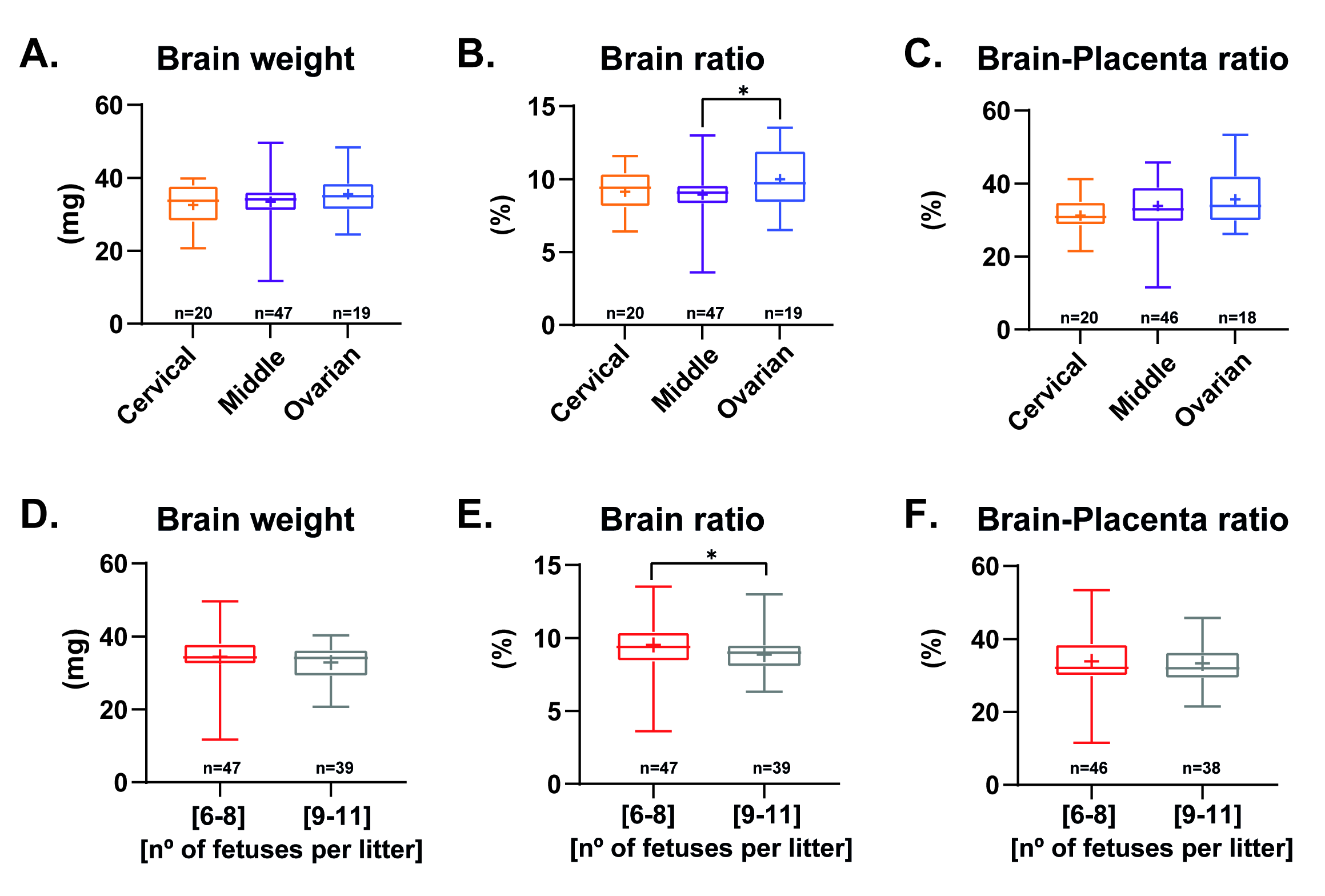

Supplement: Supplementary file 1 [file Image_1.tif]
