# Supplementary material for: Characterization of placental endocrine function and fetal brain development in a mouse model of small for gestational age
Source: Front Endocrinol (Lausanne). 2023 Feb 10;14:1116770. doi: 10.3389/fendo.2023.1116770 (PMC9950515; doi:10.3389/fendo.2023.1116770)
Supplement: Supplementary Figure 1 — Brain phenotype based on the intrauterine position and litter size. Data are from 90 fetuses in total from 11 litters (excluding outliers). Data are shown as box plots and whiskers. The rectangle shows the distribution, the line the median, the whiskers the maximum and minimum and the “+” is the mean of the group. Statistical analysis performed by one-way ANOVA followed by Bonferroni post hoc test (variables of three groups), paired or unpaired t-test (Student t-test and Mann-Whitney test, respectively). *P<0.05. [file DataSheet_1.docx]

**Supplementary Table 1. List of primers used for the expression of genes quantified in placental endocrine zone and fetal brain (Fw: forward 5′-3′; Rv: reverse 3′-5′).**

| **Gene name** | **Primer sequences** |
| --- | --- |
| *Gapdh* | Fw: CCTTCCGTGTTCCTACCCC  Rv: CCTGCTTCACCACCTTCTTG |
| *Ywhaz* | Fw: AAACAGCTTTCGATGAAGCCA  Rv: CATCTCCTTGGGTATCCGATGT |
| *Actb* | Fw: GGCTGTATTCCCCTCCATCG  Rv: CCAGTTGGTAACAATGCCATGT |
| *Igf2* | Fw: CTTGTTGACACGCTTCAGTTTG  Rv:GGGTGGCACAGTATGTCTCC |
| *H19* | Fw: CATTCTAGGCTGGGGTCAAA  Rv: GCCCTTCTTTTCCATTCTCC |
| *Hsd3b1* | Fw: GTCATTCCCAGGCAGACCAT  Rv: CTGTTCCTCGTGGCCATTCA |
| *Stard1* | Fw: TCCTCGCTACGTTCAAGCTG  Rv: CGTCGAACTTGACCCATCCA |
| *Cyp11a1* | Fw: GTCGAGATCCGGGCTTCTTT  Rv: GTCATCTCCAGCTCCGCAAT |
| *Cyp17a1* | Fw: TGGAGGCCACTATCCGAGAA  Rv: CACATGTGTGTCCTTCGGGA |
| *Prl3a1* | Fw: TGGCTCAGTACATCTCAAACCT  Rv: TTGTTCAGTGCTTGCAGGAG |
| *Prl3b1* | Fw: GGCTGCTCTTCCACATGTACC  Rv: TCACTTGCAACAGCTCCTGG |
| *Prl8a1* | Fw: ACAGATGAGGAAGGCTGCAT  Rv: TGTGTGAAGTGTCTGGAAGAGA |
| *Psg17* | Fw: CAGGTGTACTCCTCTCTTTTCATCT  Rv: TGCTCCTTTGTACCAGGTAAGT |
| *Psg18* | Fw: ACACCCTACGAACTCTGACTC  Rv: TGTCACAGCACAAGGAATGG |
| *Psg19* | Fw: GGACATCGGATTCTACACCCT  Rv: GGACAGAGTTGAAAGCGTCA |
| *Psg21* | Fw: ACGTCCACATTTCTTCAGGTC  Rv: CGTCCTCCTTCAGCAACTCT |
| *Ier3* | Fw: GATGGCGAACAGGAGAAAGAG  Rv: GCGCGTTTGAACACTTCTC |
| *Klf2* | Fw: CTTCCAGCCGCATCCTTC  Rv: GCAAGACCTACACCAAGAGC |
| *Egr1* | Fw: GATAACTCGTCTCCACCATCG  Rv: AGCGCCTTCAATCCTCAAG |
| *Ms4a7* | Fw: GTTCTCCCAGGAGCAGAGTG  Rv: GCCAGGGATGCTGTCCTC |
| *Ccr1* | Fw: TACTCTGGAAACACAGACTCACT  Rv: ACAGCAGTCTTTTGGCATGG |
| *Mrc1* | Fw: TTCAGCTATTGGACGCGAGG  Rv: GAATCTGACACCCAGCGGAA |
| *Plxna3* | Fw: TAACACATGCCAGGGCAAGAA  Rv: TGTCTGGCTTGGGGAATCAC |
| *Slit1* | Fw: TAGCATGCACTCACACCTGG  Rv: CTGCTCCCCGGATATGAACC |
| *Gabra1* | Fw: ATGTTCTAGCAGGGAAGCGAG  Rv: GAGGGCTGTCCATAGCTTCTTC |
| *Sv2b* | Fw: ACAGGCTCCGTTTAAAGGCTAT  Rv: AGGCTTGTGCTGGGAGTAAC |
| *Gabrg1* | Fw: AAACAAGACTTCGGCTTCCCC  Rv: GCCCTCCAAACACTGGTAGC |
| *Gabrg2* | Fw: CGGGCATGAATAAAATGACGCT  Rv: TTTTGGCTTGTGAAGCCTGG |
| *Lrrc4c* | Fw: TGCTGTTGCTGAAGGATCAA  Rv: ACACTTCATTTGGTTTCTTCTCAG |
| *Nrcam* | Fw: CGCTGGATGTTCCTCTCGAT  Rv: AGTCCAGGAAAAGCTTGGGG |
| *Cd9* | Fw: GCTGGGATTGTTCTTCGGGT  Rv: GGGTTCATCCTTGCTCCGTA |
| *Prlr1* | Fw: GGATTTTACACGGGGCTCAGG  Rv: CAATAGATCAGAGGCTCCCTTCAG |
| *Ascl2* | Fw: GAGCAGGAGCTGCTTGACTT  Rv: CAGTCAGCACTTGGCATTTG |
| *Tfap2c* | Fw: CCTGCTCAGCTCCACGTC  Rv: CCTCCATTTTTGGACTTTGC |
| *Peg3* | Fw: AGCACATCCCACTGTACGAA  Rv: TCTGCATTTGACCCCTGGAT |
| *Igf1r* | Fw: GTGGGGGCTCGTGTTTCTC  Rv: GATCACCGTGCAGTTTTCCA |
| *Igf2r* | Fw: CCAGTTATGCCTGCCCAGAA  Rv: GGCCGACACACATTGAGGTA |
| *Slc2a1* | Fw: GCTTATGGGCTTCTCCAAACT  Rv: GGTGACACCTCTCCCACATAC |
| *Slc2a3* | Fw: GATCGGCTCTTTCCAGTTTG  Rv: CAATCATGCCACCAACAGAG |

**Supplemetary Table 2. List of litters with the number of fetuses and presence or absence of LGA (large for gestational age) and SGA (small for gestational age)**

| Litter  ID | Total litter size | Number of LGA | Number of SGA |
| --- | --- | --- | --- |
| 1 | 7 | 0 | 2 |
| 2 | 10 | 6 | 1 |
| 3 | 8 | 0 | 0 |
| 4 | 8 | 4 | 0 |
| 5 | 10 | 0 | 0 |
| 6 | 7 | 0 | 1 |
| 7 | 9 | 1 | 0 |
| 8 | 8 | 0 | 0 |
| 9 | 6 (one fetus was found dead) | 0 | 2 |
| 10 | 11 | 0 | 3 |
| 11 | 7 | 0 | 0 |

**Supplementary Table 3. Comparison of LGA (large for gestational age) and SGA (small for gestational age) based on the number of fetuses per litter.** Statistical analysis performed by Student t-test or Mann-Whitney test based on the normality of the variable assessed by the Shapiro Wilk test. *P<0.05, **P<0.01, NS not significant.

| **Large for gestational age fetuses (LGA)** | | | |
| --- | --- | --- | --- |
|  | **LGA in litters of**  **6-8 fetuses**  (N=5) | **LGA in litters of**  **9-11 fetuses**  (N=6) | **P value** |
| **Fetal weight (mg)** | 428±9.21 | 414±2.20 | NS |
| **Placental weight (mg)** | 115±11.1 | 100±17.7 | NS |
| **F:P ratio** | 3.76±0.21 | 4.27±0.37 | NS |
| **Brain weight (mg)** | 34.9±0.61 | 37.8±1.09 | 0.050 |
| **Brain ratio (%)** | 8.18±0.10 | 9.13±0.22 | ** |
| **Brain:Placenta ratio** | 30.7±1.48 | 42±4.31 | * |
| **Small for gestational age fetuses (SGA)** | | | |
|  | **SGA in litters of**  **6-8 fetuses**  (N=5) | **SGA in litters of**  **9-11 fetuses**  (N=4) | **P value** |
| **Fetal weight (mg)** | 302±17.5 | 296±11.6 | NS |
| **Placental weight (mg)** | 102±4.76 | 96.3±2.57 | NS |
| **F:P ratio** | 2.97±0.12 | 3.08±0.11 | NS |
| **Brain weight (mg)** | 32.3±7.28 | 28.6±3.67 | NS |
| **Brain ratio (%)** | 9.10±2.02 | 9.63±1.15 | NS |
| **Brain:Placenta ratio** | 26.2±5.07 | 30±4.57 | NS |
